# Nitrate of Silver in Membranous Croup

**Published:** 1848-02

**Authors:** L. Aldrich

**Affiliations:** Reading, Vt.


13. *Nitrate of Silver in Membranous Croup.* To the Editor of the Boston Medical and Surgical Journal.

*Dear Sir*,—The reading of the case of membranous croup, treated with nitrate of silver, reported by Dr. C. E. Ware in your Journal, No. 21. vol. 37, has induced me to send you the following case, which came under my observation, for insertion in the Journal, if you think best.

On the 23d of November last, I was requested to visit H. P., aged about 15 years, laboring, as her parents supposed, under a severe and protracted cold. Her disease, in fact, was *cynanche trachealis*, evidently, in my mind, to prove fatal soon, unless something could be done to remove the *false membrane* which had been formed, nobody knows how long. It was a case characteristic of croup in its last stages; face flushed and swollen; eyes protuberant; breathing was performed with a frightful hissing noise; pulse 110 in a minute. Gave her an emetic immediately, which gave temporary relief. Ordered onion poultice to the neck, and prescribed such other medicines as in my judgment were called for. This was in the evening. Visited her the next morning. As I anticipated, I found her no better. Could hear her breathe, although in an adjoining room with closed doors. Realizing sensibly, that her case would prove fatal, unless some more efficient means could be devised, I resolved, as a *dernier resort*, to make use of a strong solution of nitrate of silver. It was accordingly prepared, and a spongy substance, well saturated with it, was introduced low down into the trachea. The breathing presently grew worse; but within an hour looseness seemed to take place, which promised relief. Considerable slimy, ropy matter was got rid of, which, as the saying is, seemed to come from the "right spot," and within an hour and a half from the time the solution was made use of, a piece of false membrane was thrown off, an inch long, hollow, tube-like. The effect was immediate relief. Her breathing, which was distressingly performed but a short time previously, was now nearly natural, and she could talk distinctly, which she had not done for many days before.

So much for the nitrate of silver in this case. What it will do in all similar cases, I cannot say. At any rate, if one presents itself, which I sincerely pray never will, I shall most assuredly give it a fair trial. In conclusion, I would say, that I have not the least doubt that it saved the life of the patient above referred to. L. ALDRICH.

Reading, Vt., Feb. 1848.—*Ibid.*

